# Semi-Continuous Fermentation of Onion Vinegar and Its Functional Properties

**DOI:** 10.3390/molecules22081313

**Published:** 2017-08-08

**Authors:** Sulhee Lee, Jin-A Lee, Gwi-Gun Park, Jae-Kweon Jang, Young-Seo Park

**Affiliations:** 1 Department of Food Science and Biotechnology, Gachon University, Seongnam 13120, Korea; sulhee2340@gmail.com (S.L.); gene_a0963@naver.com (J.-A.L.); ggpark@gachon.ac.kr (G.-G.P.); 2 Food Nutrition Major, School of Food, Chungkang College of Cultural Industries, Icheon 17390, Korea; jkjang@chungkang.ac.kr

**Keywords:** semi-continuous fermentation, vinegar, onion, *Acetobacter orientalis*, *Saccharomyces cerevisiae*, two-stage fermentation

## Abstract

For the fermentation of vinegar using onion, acetic acid bacteria and yeast strains with high fermentation ability were screened. Among them, *Saccharomyces cerevisiae* 1026 was selected as a starter for ethanol production and *Acetobacter orientalis* MAK88 was selected as a vinegar producer. When the two-stage fermentation of onion vinegar was performed at 28 °C, the titratable acidity reached 4.80% at 24 h of fermentation. When semi-continuous fermentation proceeded to charge-discharge consisting of three cycles, the acetic acid content reached 4.35% at 48 h of fermentation. At this stage, the fermentation efficiency, acetic acid productivity, and specific product formation rate were 76.71%, 17.73 g/(L·d), and 20.58 g/(g·h), respectively. The process in this study significantly reduced the fermentation time and simplified the vinegar production process. The content of total flavonoids and total polyphenols in onion vinegar were 104.36 and 455.41 μg/mL, respectively. The antioxidant activities of onion vinegar in terms of 2,2-diphenyl-1-picrylhydrazyl (DPPH) radical scavenging activity, 2,2′-azino-bis(3-ethylbenzothiazoline-6-sulphonic) acid (ABTS^+^) radical scavenging activity, and reducing power were 75.33%, 98.88%, and 1.28, respectively. The nitrite scavenging abilities of onion vinegar were 95.38 at pH 1.2. The onion vinegar produced in this study showed higher organoleptic acceptability than commercial onion vinegar.

## 1. Introduction

Vinegar is a typical fermented food with a history of more than 10,000 years; it is produced by biological transformation via microorganisms [1]. In Korea, vinegar has long been produced using grains, but the traditional fermentation of vinegar is time-consuming. Since the 1950s, extensive efforts have been made to reduce the fermentation period of commercially produced vinegar [2]. Many fermentation processes have been developed to increase the rate of biological reactions that aerobically transform ethanol into acetic acid for the industrial production of vinegar [3].

Vinegar fermentation usually takes place in two stages, i.e., the facultative anaerobic conversion of glucose to ethanol, and the aerobic oxidation of ethanol to acetic acid [3]. In the ethanol oxidation step, acetic acid bacteria convert ethanol to acetic acid via acetaldehyde [4]. This oxidation process is important for aerobic fermentation by two sequential reaction mechanisms using membrane-bound alcohol dehydrogenase and aldehyde dehydrogenase linked to the respiratory chain [5]. At the end of acetification, acetic acid bacteria are resistant to acetic acid, but begin to assimilate acetate and CO_2_ via the tricarboxylic acid (TCA) cycle. This phenomenon is called ‘overoxidation’ and can limit the fermentation of acetic acid [6].

Vinegar contains abundant amino acids, organic acids, and certain minerals and is not only used as condiment and food preservative [1,7], but also has various beneficial effects, e.g., digestive and appetite stimulant, antioxidant, fatigue recovery, lipid lowering effects, and blood pressure regulation [8]. Vinegar can be produced using a variety of ingredients, e.g., Chinese and Japanese cereal vinegar, French wine vinegar, British malt vinegar, Southeast Asian persimmon, and pineapple vinegar [9,10].

Onions are the world’s third most produced vegetable after tomatoes and watermelons owing to its excellent climate adaptability [11]. In Korea, approximately 1,500,000 tons of onions are produced per year. However, about 15% of harvested onions are usually disposed of as agricultural waste because they fail to meet quality standards [11]. Therefore, it is necessary to increase the value of onions by processing. Onions are rich in flavonoids, such as quercetin, sulfur compounds, and alcohol propyl disulfide, which confer health benefits in humans. Quercetin, the main flavonoid in onions, can prevent cancer and heart disease and alleviate phenomena associated with aging [12,13,14]. In addition, onion consumption seems to have a beneficial effect in improving the diabetic state [15]. Despite the many advantageous properties of onions, they do not have favorable organoleptic properties, owing to their unique bitter taste; this can be improved by fermentation [16,17].

In general, the most common process for onion fermentation in the food industry is based on the submerged culture method; however, in this study, a semi-continuous culture method, which has received increasing industrial interest, was used [18]. In this process, a portion of the total volume of the fermentation medium is discharged at the completion of a fermentation cycle, and the remaining medium is replenished with the same volume of fresh medium to obtain the final working volume [19]. Then, a new fermentation cycle begins. In this study, optimum fermentation conditions for onions were examined using a two-stage semi-continuous fermentation process for the production of onion vinegar with high efficiency. The functional properties of onion vinegar were then estimated.

## 2. Results and Discussion

### 2.1. Screening of Acetic Acid Bacteria and Yeast

A total of 161 strains isolated from fallen fruits and fermented foods showed large transparent zones around colonies on YCE agar plates. Among these strains, strain No. MAK88, which was isolated from Korean traditional rice wine, exhibited high acid production (3.51%) and a good growth rate when cultured in onion juice, and thereby was selected as a starter for acetic acid fermentation. Resistance to acid or alcohol during fermentation is an important property for a good starter, because acid or alcohol produced during fermentation might hamper the growth and thereby the fermentation ability of the starter. Strain No. MAK88 also showed high acid- and alcohol-tolerant properties when compared with those of the other isolates. This strain was identified as *Acetobacter orientalis* MAK88 based on a phylogenetic tree of the nucleotide sequence of the 16S ribosomal RNA (rRNA) gene.

Ethanol production by 47 strains of *Saccharomyces cerevisiae* obtained from the Korean Culture Collection of Probiotics was evaluated. Finally, *S. cerevisiae* 1026, which was isolated from *Nuruk* (a Korean traditional fermentation starter made of dry ground rice and wheat), was chosen for ethanol fermentation based on its high ethanol production (9.51%) using onion juice as a fermentation medium.

### 2.2. Two-Stage Onion Vinegar Fermentation

For the fermentation of onion vinegar, onion juice was fermented using *S. cerevisiae* 1026 to produce ethanol, the substrate for acetic acid fermentation. This fermentation broth was used for acetic acid fermentation using *A. orientalis* MAK88. Because the oxygen supply rate to the medium is an important parameter in vinegar fermentation, cell growth and ethanol or acetic acid production according to aeration (in terms of vvm) were determined.

**Figure 1:**
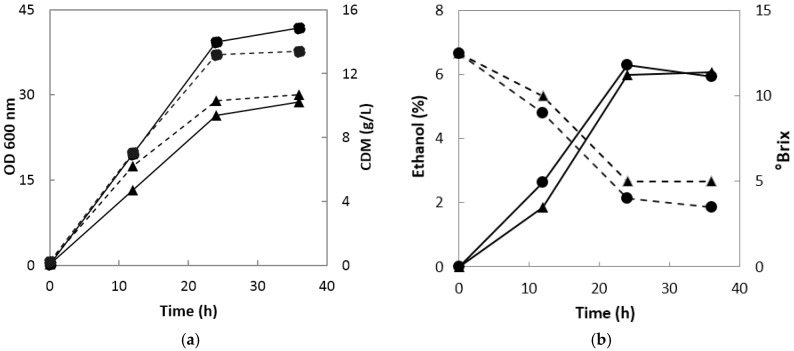
Growth of *S. cerevisiae* 1026 and changes in ethanol production and °Brix during ethanol fermentation. (**a**) Absorbance at a wavelength of 600 nm and cell dry mass (CDM); solid line, optical density (OD) 600 nm; dotted line, CDM; ▲, aeration with 0.1 vvm; ●, aeration with 0.5 vvm (**b**) Ethanol production and °Brix. Solid line, ethanol concentration; dotted line, °Brix; ▲, aeration with 0.1 vvm; ●, aeration with 0.5 vvm.

When onion juice was fermented using *S. cerevisiae* 1026, the absorbance of the fermentation broth and cell dry mass increased sharply up to 24 h for both 0.1 and 0.5 vvm, but remained almost unchanged thereafter (Figure 1a). The absorbance and cell dry mass at 0.5 vvm were 1.4 and 1.3 times higher than those at 0.1 vvm, respectively. Ethanol production was 0.78% and 0.28% higher at 0.5 vvm than at 0.1 vvm at 12 and 24 h, respectively, but 0.13% higher at 0.1 vvm than at 0.5 vvm at 36 h (Figure 1b). These results confirmed that the optimal oxygen supply rate and fermentation time for ethanol production were 0.1 vvm for 24 h.

**Figure 2:**
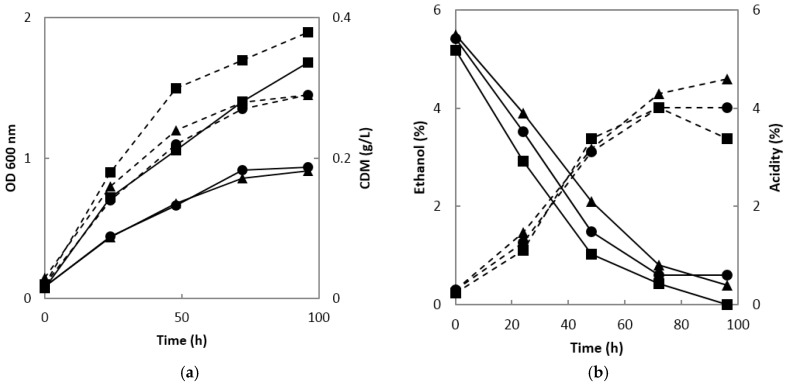
Growth of *A. orientalis* MAK88 and changes in ethanol consumption and titratable acidity during acetic acid fermentation. (**a**) Absorbance at a wavelength of 600 nm and cell dry mass (CDM); solid line, OD 600 nm; dotted line, CDM; ▲, aeration with 0.2 vvm; ●, aeration with 0.5 vvm; ■, aeration with 1 vvm; (**b**) Ethanol consumption and titratable acidity; solid line, ethanol concentration; dotted line, titratable acidity; ▲, aeration with 0.2 vvm; ●, aeration with 0.5 vvm; ■, aeration with 1 vvm.

When the ethanol fermentation broth was used for acetic acid fermentation using *A. orientalis* MAK88, the absorbances of the fermentation broth at both 0.2 and 0.5 vvm were 0.9 at 24 h of fermentation, which was substantially higher than those at 1 vvm. The cell dry mass showed a similar pattern; it was 0.29 g/L at 0.2 and 0.5 vvm and 0.38 g/L at 1 vvm (Figure 2a). Acetic acid production was similar at 0.2, 0.5, and 1 vvm up to 72 h and exceeded 4% in all conditions; however, after 72 h, the acidity decreased at 1 vvm, did not change at 0.5 vvm, and increased slightly at 0.2 vvm. De Ory et al. [20] reported the possibility of the volatilization of ethanol and acetic acid during the fermentation of acetic acid. In this study, as the air injection rate increased, the volatilization of ethanol increased. In addition, at 0.5 and 1 vvm, substantial air bubbles were generated due to the large amount of air injected, necessitating an antifoaming agent. However, no antifoaming agent was required at 0.2 vvm. Saeki [21] used the same air injection rate of 0.2 vvm used in this study, but acetic acid production in most fermenter experiments was maximized at 1 vvm [22,23] (Figure 2b).

The estimated fermentation yield (Y_A/E_) was 65.47% at 0.2 vvm, 5.14% and 4.38% higher than those at 0.5 and 1 vvm, respectively. The productivity of acetic acid (P) was 10.75, 9.30, and 9.45 g/(L·d) at 0.2, 0.5 and 1 vvm, respectively, indicating that productivity was highest at 0.2 vvm. In addition, the specific production rate (Π) of acetic acid was 18.88 g/(g·h) at 0.2 vvm, twice as high as that at 1 vvm (Table 1).

Therefore, considering the conversion yield, volumetric and specific productivity of acetic acid, our results indicated that acetic acid fermentation with an air injection rate of 0.2 vvm was most suitable.

### 2.3. Semi-Continuous Acetic Acid Fermentation Using a Fermenter

Semi-continuous fermentation proceeded to charge-discharge cycles by removing 75% of the acetic acid fermentation broth and replacing it with an equal volume of fresh substrate; three cycles were used, with an initial acetic acid concentration of 1% and initial ethanol concentration of 4–5%.

**Figure 3:**
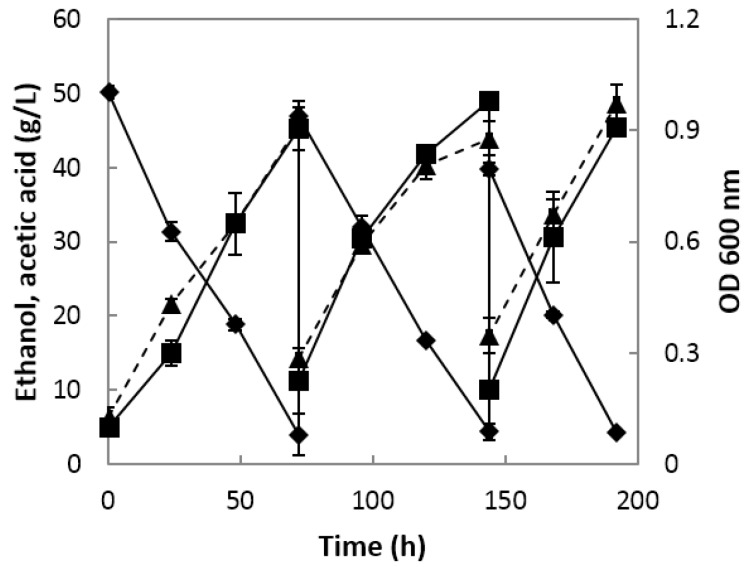
Ethanol consumption, titratable acidity, and growth rate of *A. orientalis* MAK88 by semi-continuous onion vinegar fermentation using a fermenter. ◆, alcohol concentration; ■, acetic acid concentration; ▲ with dotted line, cell growth (OD 600 nm).

As semi-continuous fermentation progressed, the bacterial cells gradually increased and were highest on the second day of the third cycle, with an absorbance of 0.974 (Figure 3). The first cycle had an initial ethanol concentration of 5% and was terminated when the residual ethanol content was 0.4%, and the final acidity was 4.53%. The second cycle was carried out until the residual ethanol content was 0.44% and the acidity was 4.9%. In the third cycle, the initial ethanol content was lowered by 1% from the previous cycle to lower the residual ethanol content on the second day. On the second day of the third cycle, the acidity was more than 4% and the alcohol content was 0.42%.

In cycle 3, the fermentation yield (Y_A/E_), acetic acid productivity (P), and specific production rate (Π) of acetic acid were the highest, i.e., 76.71%, 17.73 g/(L·d), and 20.58 g/(g·h), respectively (Table 1). Cycles 1 and 2 showed very similar values, i.e., 66.97% and 67.81%, for fermentation yield (Y_A/E_), 13.41 and 12.53 g/(L·d) for acetic acid productivity (P), and 18.57 and 18.80 g/(g·h) for the specific production rate (Π) of acetic acid, respectively (Table 1).

De Ory et al. [20] performed semi-continuous fermentation with a 50% charge-discharge system by adjusting the initial ethanol concentration and acetic acid concentration to 4–5% (*v*/*v*) and 2–5% (*v*/*v*), respectively, using wine as a substrate for vinegar fermentation. The fermentation time was 1 day longer than that in this study, and acetic acid productivity was 11.1 g/(L·d), which was lower than the acetic acid productivity observed in this study. These results suggest that the process used in this study is beneficial for industrial fermentation.

Qi et al. [19] used semi-continuous fermentation after adjusting the initial acetic acid concentration to 3%, 4%, 4.5%, 5% and 5.5% (*v/v*), and found that the fermentation rate and efficiency decreased when the initial acetic acid concentration exceeded 5%. When the initial acetic acid concentration was 3% (*v*/*v*), the fermentation yield and acetic acid productivity were the highest at 91.84% and 1.85 g/(L·h), respectively.

Horiuchi et al. [7] adapted a two-step fermentation system combining a repeated batch process using a charcoal pellet bioreactor and observed a maximum productivity and acetic acid yield of 3.3 g/(L·h) and 86%, which were higher than those obtained in this study. The two-step semi-continuous fermentation process used in this study was simpler and easier to operate than the system used by Horiuchi et al. [7] and produces onion vinegar in a very short time; therefore, it is more appropriate for the commercial production of onion vinegar.

### 2.4. Quality Characteristics Analysis of Onion Vinegar

The analysis of total flavonoids, total polyphenols, antioxidant activity, nitrite scavenging activity, and organic acid content and sensory evaluation of onion vinegar prepared by ethanol and acetic acid fermentation using *S. cerevisiae* 1026 and *A. orientalis* MAK88, respectively, were carried out.

#### 2.4.1. Total Flavonoid Contents

**Figure 4:**
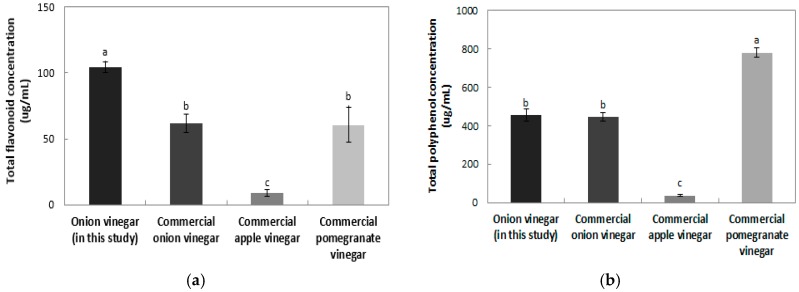
Total flavonoid and total polyphenol contents of onion vinegar in this study and various commercial vinegars. (**a**) Total flavonoid content; (**b**) Total polyphenol content. Different letters are indicative of a statistically significant difference (*p* < 0.05).

The total flavonoid contents of onion vinegar and various commercial vinegars are shown in Figure 4a. The flavonoid content of the onion vinegar developed in this study, was the highest at 104.36 μg/mL. Commercial onion vinegar, pomegranate vinegar, and apple vinegar contained 61.64, 60.65, and 9.02 μg/mL, respectively. Na et al. [24] reported that the total flavonoid contents of commercial persimmon vinegar, fig vinegar, brewing vinegar, rice vinegar, and apple vinegar samples were 194.85, 91.75, 86.05, 17.41, and 9.41 mg/kg, with apple vinegar showing similar results to those of this study. All vinegars, except for persimmon vinegar, were found to contain a lower flavonoid content than the onion vinegar produced in this study, demonstrating its high flavonoid content when compared to most other vinegars. Difference in the total flavonoid contents between the onion vinegar developed in this study and commercial onion vinegar might have resulted from differences in the preparation method of onion juice or in the variety of onion used.

#### 2.4.2. Total Polyphenol Contents

The total polyphenol content in onion vinegar and various commercial vinegars is shown in Figure 4b. The polyphenol content in commercial onion vinegar, pomegranate vinegar, and apple vinegar was 446.80, 780.47 and 37.43 μg/mL, respectively, and the polyphenol content of onion vinegar developed in this study was 455.41 μg/mL, the second highest polyphenol concentration. Na et al. [24] showed that total polyphenol content of commercial persimmon, fig, brewed, rice, and apple vinegar samples were 485.13, 320.94, 284.10, 83.86 and 41.97 mg/kg respectively. The total polyphenol content of the onion vinegar produced in this study was lower than that of persimmon vinegar. Hong et al. [25] reported that the total polyphenol content of bokbunja (*Rubus coreanus*) vinegar was 251.90 μg/mL, indicating a lower polyphenol content than that of the onion vinegar produced in this study.

#### 2.4.3. Antioxidant Effects

**Figure 5:**
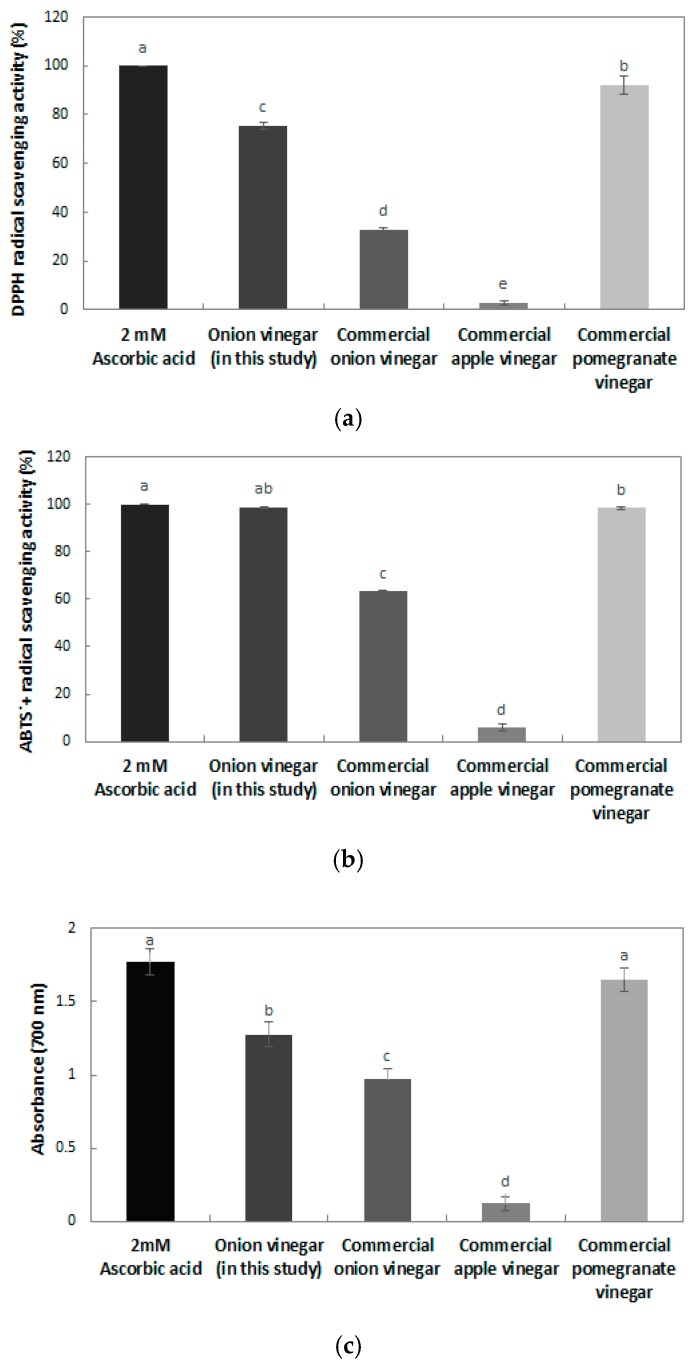
DPPH free radical scavenging activity, ABTS^+^ free radical scavenging activity, and reducing power effects of onion vinegar produced in this study and various commercial vinegars. (**a**) DPPH free radical scavenging activity; (**b**) ABTS^+^ free radical scavenging activity; (**c**) Reducing power effects. Different letters are indicative of a statistically significant difference (*p* < 0.05).

The 2,2-diphenyl-1-picrylhydrazyl (DPPH) free radical scavenging activity of the onion vinegar produced in this study and commercial vinegar is shown in Figure 5a. Compared with the 2 mM ascorbic acid positive control, onion vinegar had a 75.33% scavenging ability, and commercial pomegranate vinegar had the greatest DPPH free radical scavenging ability (92.13%). Commercial onion vinegar and apple vinegar showed 33.05 and 2.91% DPPH free radical scavenging abilities respectively, which was low. The DPPH radical scavenging activity of bokbunja vinegar and omija vinegar was 65% [26] and 65.5% [27], respectively, a lower scavenging activity than that of the onion vinegar in this study. According to the report of Kim et al. [28], the DPPH radical scavenging activity of commercial apple, plum, and lemon fruit vinegars produced with only acetic acid fermentation ranged between 16.37% and 35.76%. However, apple, plum, and red grape vinegars produced with two stages of alcohol and acetic acid fermentation were found to have a higher DPPH radical scavenging ability, ranging between 59.66 and 65.99.

The 2,2′-azino-bis(3-ethylbenzothiazoline-6-sulphonic) acid (ABTS^+^) radical scavenging activity of the vinegar is also shown in Figure 5b. Compared with the 2 mM ascorbic acid positive control, onion vinegar showed the highest ABTS^+^ radical scavenging ability (98.88%), and commercial pomegranate, onion, and apple vinegar had a 98.43, 63.27 and 5.85% ABTS^+^ radical scavenging ability, respectively. The ABTS^+^ radical scavenging ability of commercial apple vinegar was ranged between 11.78% and 99.61% [29], indicating that there was a difference in ABTS^+^ radical scavenging activity depending on the raw materials. Compared with commercial vinegars, the vinegar of this study had a high ABTS^+^ radical scavenging activity. The ABTS^+^ radical scavenging activity of these vinegars tends to be somewhat higher than that of the DPPH radical scavenging activity. As reported by Wang et al. [30], DPPH is a free radical, but ABTS^+^ is a cationic radical. It seems that there are differences in the types of phenol compounds bonded to the radicals.

Reducing power is directly related to antioxidant activity, and it can be confirmed that the onion vinegar developed in this study had antioxidant properties [31]. The reducing power measurement results for the onion vinegar produced in this study and commercial vinegars are shown in Figure 5c. The 2 mM ascorbic acid control showed the highest reducing power of 1.77, and commercial pomegranate vinegar and non-commercial onion vinegar showed reducing powers of 1.65 and 1.28, respectively. The reducing power of vinegar is reported to differ according to the raw material. In the case of bokbunja vinegar, the reducing power was 0.50 at a concentration of 500 μg/mL [26], and the reducing power of vinegar containing 7% Akebia berry was reported to be 1.29 [32]. Extracts with high DPPH radical scavenging activity and ABTS^+^ radical scavenging ability were also reported to have high reducing power, indicating that the antioxidant effect of onion vinegar developed in this study was high.

#### 2.4.4. Nitrite Scavenging Ability

Table 2 shows the nitrite scavenging activity of the onion vinegar and the commercial vinegars after adjusting pH to 1.2, 3.0 and 4.2. The lower the pH, the higher the nitrite scavenging ability. Onion vinegar produced in this study had a nitrite scavenging ability of 95.38%, 49.37% and 29.32% at pH 1.2, 3.0 and 4.2, respectively, and was superior to the 10 mM ascorbic acid control at pH 3.0 and 4.2. Regarding the raw materials, it was confirmed that omija vinegar had a nitrite scavenging ability of 34% at pH 1.2 [27], and the nitrite scavenging ability of various brown rice vinegars was reported to range between 6.26% and 21.31% [33]. It was found to have a very high nitrite scavenging ability compared with the results of this study. Commercial pomegranate, onion, and apple vinegar showed the highest nitrite scavenging ability at pH 1.2, 89.68%, 61.99% and 14.62%, respectively. These results suggest that it is likely to inhibit nitrosamine production when carcinogenic nitrosamines are produced in the strongly acidic pH condition of the human body [28].

### 2.5. Organic Acid Analysis and Sensory Evaluation

*S. cerevisiae* 1026 and *A. orientalis* MAK88 strains were used for ethanol and acetic acid fermentation, respectively. Onion vinegar was prepared and the organic acid content was measured (Table 3). The main organic acid in onion vinegar was acetic acid, accounting for 3.68% out of the 4.22% total organic acid content, or 87.2% of the organic acid present. Citric acid and malic acid contents were low, being 0.45% and 0.04%, respectively, and lactic acid and succinic acid were not detected. Shin et al. [34] reported that acetic acid, succinic acid, and malic acid content was high in the organic acid content of onion vinegar fermented in two stages. It has been reported that the organic acids present in vinegar have no correlation with sensory properties, and the acetic acid to total organic acids (A/T) ratio is highly correlated with the taste [35]. In addition to onion vinegar, potato and kiwifruit vinegar were reported to have A/T ratios of 0.66 and 0.77, respectively, and A/T ratios were found to be in the range of 0.74–0.93 for commercial fruit vinegars [36]. The onion vinegar in this study showed an A/T ratio of over 0.87, which was a better result than that of other studies, so we expected a positive sensory result.

**Figure 6:**
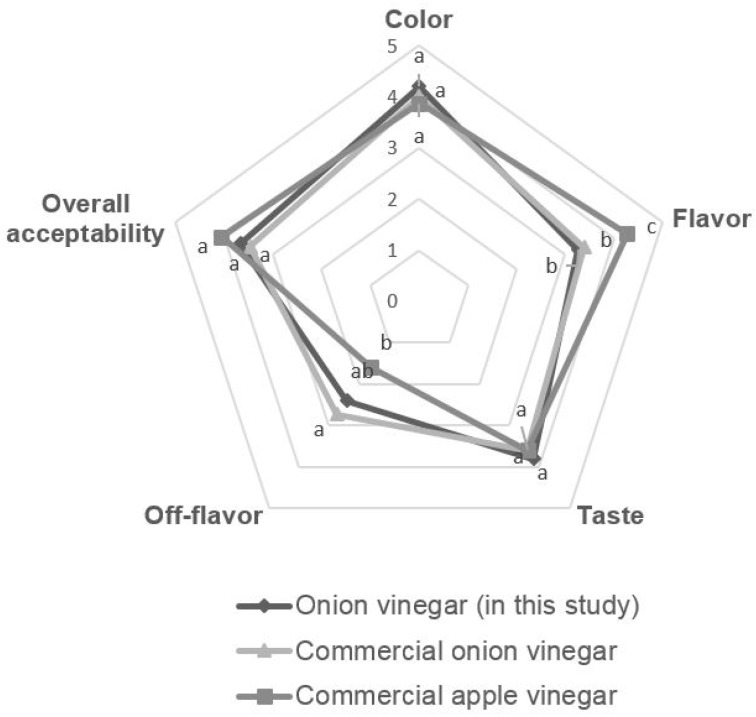
The sensory evaluation of onion vinegar in this study and various commercial vinegars. Different alphabets on the pentagonal vertices are indicative of a statistically significant difference (*p* < 0.05).

The sensory evaluations of color, flavor, taste, off flavor (fermentation odor), and overall acceptability of onion vinegar in this study and commercial vinegar are shown in Figure 6, respectively. For the color and taste preference, the onion vinegar developed in this study was the best, at 4.2 and 3.8, respectively. The flavor and off flavor (fermentation odor) were the best for commercial apple vinegar, at 4.2 and 1.6, respectively. This is because commercial apple vinegar is supplemented with apple flavoring, which should be considered when comparing the results. The overall acceptability of commercial apple vinegar, non-commercial onion vinegar, and commercial onion vinegar is 4.07, 3.67 and 3.47, respectively. The onion vinegar produced in this study showed better flavor, less off flavor (fermentation odor), and slightly better overall acceptability than commercial onion vinegar. When comparing the organic acid content, the acetic acid content of onion vinegar produced in this study was 87.41%, higher than that of commercial onion vinegar. Sensory evaluation also showed positive results based on the absence of lactic acid, which can give a negative sensory effect.

## 3. Materials and Methods

### 3.1. Selection of Acetic Acid Bacteria and a Yeast Strain

Acetic acid bacterial strains were isolated from fallen fruits collected from local orchards and fermented foods purchased from local markets in Korea. Fallen fruits and fermented foods were resuspended and serially diluted with 0.88% (*w*/*v*) NaCl, and 100 μL of diluted suspension was spread onto a YCE agar plate (3 g of glucose, 1 g of peptone, 1 g of beef extract, 4 mL of ethanol, 1 g of CaCO_3_, 1.5 g of agar, 88.5 mL of distilled water). Plates were incubated at 30 °C for 3 days and colonies surrounded by a transparent zone were as acetic acid bacteria. These bacterial strains were cultured in YCE broth (without CaCO_3_) containing 4% (*v*/*v*) ethanol at 28 °C for 72 h at 200 rpm using a shaking incubator (MMS-210; EYELA, Tokyo, Japan) and the culture broth was centrifuged at 16,000× *g* for 1 min. Strains for which the culture supernatant had a titratable acidity of 2% or more were selected as strains with high acid production. These selected strains were cultured in onion juice containing 6% (*v*/*v*) ethanol at 28 °C for 5 days at 200 rpm, and the culture supernatant with the highest titratable acidity was used to select a strain for the fermentation of onion vinegar.

Yeast strains for the fermentation of ethanol were supplied from the Korean Culture Collection of Probiotics (Seongnam, Korea). Yeast strains were cultured in onion juice at 30 °C for three days at 100 rpm, and the strain with the highest ethanol production was selected for ethanol fermentation.

### 3.2. Identification of Acetic Acid Bacteria

The molecular identification of acetic acid bacterial isolates was performed by a sequence analysis of 16S rRNA. Genomic DNAs of acetic acid bacteria were isolated using the Wizard Genomic DNA Purification Kit (Promega, San Luis Obispo, CA, USA) according to the manufacturer’s instructions. The 16S rRNA gene from acetic acid bacteria was amplified by polymerase chain reaction (PCR) using the following universal primers: 27F (5′-AGA GTT TGA TCA TGG CTC AG-3′) and 1492R (5′-AAG GAG GTG ATC CAA CCG CA-3′). PCR was conducted using the MyCycler (BIO-RAD Laboratories, Hercules, CA, USA) and PCR amplicons were subjected to a sequencing analysis (Macrogen, Daejeon, Korea). A sequence alignment was generated using a BLAST search against the National Center for Biotechnology Information (http://www.ncbi.nlm.nih.gov) and a phylogenetic tree was constructed using MEGA4 (http://www.megasoftware.net/). The nucleotide sequence of 16S rRNA gene of *Acetobacter orientalis* MAK88, which was used as vinegar producer, was deposited to GenBank database (GenBank accession No. KY855503).

Lactic acid bacteria were also identified biochemically using the API 50 CHL Kit according to the manufacturer’s instructions, and the results were interpreted using apiweb (bioMeriuex, Marcy- l’Étoile, France).

### 3.3. Analytical Methods

The growth of bacterial and yeast cells was measured as the absorbance at a wavelength of 600 nm using a spectrophotometer (BioSpec-mini; SHIMADZU, Kyoto, Japan). Samples for analysis were prepared by periodic removal from the fermentation broth and centrifugation at 13,000× *g* for 1 min, followed by collection of the supernatant. To determine the titratable acidity, 9 mL of sample was added to 10 mL of distilled water and titrated with 0.1 N sodium hydroxide (Samchun Chemical, Seoul, Korea) using phenolphthalein (Sigma-Aldrich, St. Louis, MO, USA) as an indicator. The volume of NaOH used in the titration was expressed as the titratable acidity (%) for neutralizing lactic acid as in Equation (1):

(1) $$\mathrm{Titratable}\;\mathrm{acidity}\;\left(\%\right)=\frac{0.1N\;\mathrm{NaOH}\;\left(\mathrm{mL}\right)\times 0.009\times 100}{sample\;weight\;\left(g\right)}$$

where, 0.009 was lactic acid equivalent.

The pH and sugar content (°Brix) were determined using a pH meter (FE20; Mettler Toledo, Columbus, OH, USA) and refractometer (MT-325; Atago Co., Ltd., Tokyo, Japan), respectively. The ethanol concentration was determined using the Roche Ethanol Assay Kit (Cat. No. 10 176 290 035; Roche, Basel, Switzerland) according to the manufacturer’s protocol.

### 3.4. Preparation of the Onion Extract

Onions were purchased at a local market in Korea, and onion juice was prepared by heating whole onions at 121 °C for 15 min, with squeezing, filtering, and sterilization at 121 °C for 15 min.

### 3.5. Two-Stage Onion Vinegar Fermentation

#### 3.5.1. Ethanol Fermentation

After the sugar concentration of the onion juice was adjusted to 12 °Brix by adding sucrose, the seed culture of *S. cerevisiae* 1026 was inoculated at 1% (*v*/*v*). Fermentation of onion juice was conducted using a stirred tank fermenter equipped with a six-bladed flat impeller (model No. KF-5L, Korea Fermentation Co., Ltd., Seoul, Korea). Fermentation was carried out at 28 °C for 36 h at 100 rpm with a 0.1 or 0.5 vvm oxygen supply rate in a glass 5-L fermentation vessel with a 2.5-L working volume. The cell growth rate, ethanol production, and changes in °Brix were monitored. The growth of the strain was measured based on the absorbance at 600 nm and dry weight at 80 °C.

#### 3.5.2. Acetic Acid Fermentation

The ethanol fermentation broth of the onion juice was centrifuged at 16,100× *g* for 20 min, filtered through a 0.45-μm membrane filter (Millipore, Billerica, MA, USA), and supplemented with unfermented onion juice to adjust the ethanol concentration to 5% (*v*/*v*). The seed culture of acetic acid bacteria was inoculated at 5% (*v*/*v*) in ethanol fermentation broth, and fermentation was performed in a 2.5-L working volume with various oxygen supply rates (0.2, 0.5 and 1 vvm) at 28 °C for 96 h at 300 rpm. The titratable acidity of the fermentation broth and ethanol consumption were examined.

#### 3.5.3. Semi-Continuous Acetic Acid Fermentation Using a Fermenter

Sucrose was added to the onion juice to adjust the sugar concentration to 12 °Brix, and the seed culture of *S. cerevisiae* 1026 was inoculated at 1%, followed by fermentation at 28 °C for 12 h at 100 rpm with an oxygen supply of 0.1 vvm. The fermentation broth was centrifuged at 16,100× *g* for 20 min and filtered through a 0.45-μm membrane filter (Millipore). The onion juice was added to the fermentation broth to adjust the ethanol concentration to 5% (*v*/*v*), the seed culture of acetic acid bacteria was inoculated, and the start-up fermentation was performed at 28 °C, 300 rpm, and an oxygen supply of 0.2 vvm.

**Figure 7:**
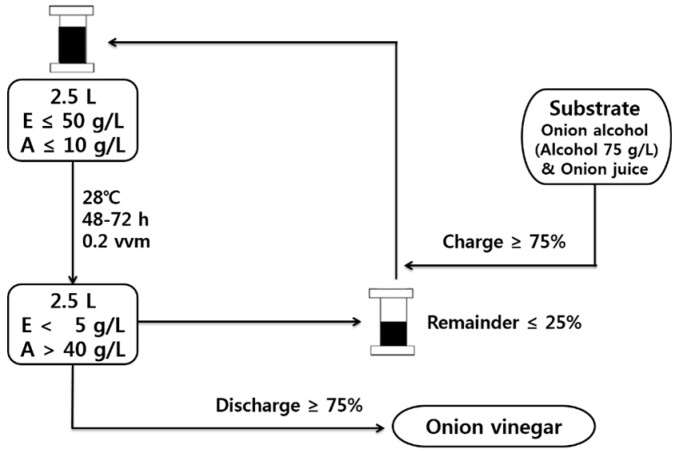
Schematic diagram of the semi-continuous fermentation process. E represents ethanol concentration and A represents acetic acid concentration.

The semi-continuous fermentation proceeded to charge-discharge, which was carried out by removing 75% of the acetic acid fermentation broth and adding the same amount of ethanol-fermented onion juice for 3 cycles, with an initial acetic acid concentration of 1% and an initial ethanol concentration of 4–5% (Figure 7).

### 3.6. Total Flavonoid Content

Total flavonoid content was determined by adding 1.8 mL of ethanol, 0.1 mL of 10% aluminum chloride (AlCl_3_), 0.1 mL of 1 M potassium acetate, and 2.8 mL of distilled water to 0.5 mL of the sample extract according to the method of Zhishen et al. [37] and Zou et al. [38]. Samples were mixed in this order, and the absorbance was measured at 415 nm using a spectrophotometer (BioSpec-mini, Shimadzu, Japan). As a standard material, quercetin (Sigma-Aldrich, St. Louis, MO, USA) was prepared at a concentration of 0–200 μg/mL and the total flavonoid content was measured from the standard calibration curve obtained by the same method.

### 3.7. Total Polyphenol Content

Analysis of the total polyphenol compounds was carried out according to the Folin-Denis method [39]. A volume of 1.8 mL distilled water and 0.2 mL 2 N Folin-Ciocalteu’s phenol reagent was added to 0.2 mL of the sample, mixed with 2 mL of 7% (*w*/*v*) sodium carbonate (Na_2_CO_3_) for 6 min, and allowed to stand for 90 min. The absorbance was measured at 730 nm using a spectrophotometer (BioSpec-mini, Shimadzu, Japan). The total sample phenol content was determined from a 0–200 μg/mL gallic acid (Sigma-Aldrich) standard calibration curve.

### 3.8. DPPH Radical Scavenging Activity

The electron donating ability of the sample was measured according to the method of Blois [40] and the DPPH hydrogen donating effect was measured. A 0.27 mL volume of 0.5 mM DPPH solution (in methanol) was added to 0.03 mL of the sample, followed by incubation for 20 min. The absorbance was then measured at 517 nm using a spectrophotometer (Shimadzu). Electron donating ability [EDA (%)] was calculated using 2 mM ascorbic acid as a positive control.

(2) $$\mathrm{EDA}\;\left(\%\right)=\left(1-\left(\frac{Asample}{Acontrol}\right)\right)\times 100$$

where, A*sample*: absorbance of the sample added group; A*control*: absorbance of the no sample added group.

### 3.9. ABTS^+^ Radical Scavenging Activity

ABTS^+^ radical scavenging activity was measured by modifying the method of Biglari et al. [41]. 2,2′-azobis (2-amidinopropane) dihydrochloride (7 mM, Sigma-Aldrich) was mixed with 2.45 mM potassium peroxodisulfate and reacted in the dark for 16 h. The absorbance of ABTS^+^ solution was adjusted at 734 nm to be 1.00 ± 0.005, and then 0.1 mL of sample and 3.9 mL of ABTS^+^ solution were mixed and the absorbance was measured at 734 nm after reaction for 6 min. The positive control group was 2 mM ascorbic acid. The ABTS^+^ radical scavenging activity was calculated according to Equation (3).

(3) $${\mathrm{ABTS}}^{+}\;\mathrm{scavenging}\;\mathrm{activity}\;\left(\%\right)=\left(1-\left(\frac{Asample}{Acontrol}\right)\right)\times 100$$

where, A*sample*: absorbance of the sample added group; A*control*: absorbance of the no sample added group.

### 3.10. Reducing Power Measurement

The reducing power of the samples was measured by modifying the method of Yildirim et al. [42]. A 2.5 mL volume of 0.2 M phosphate buffer solution (pH 6.6) and 2.5 mL of 1% (*w*/*v*) potassium ferricyanide were added to 2.5 mL of the sample, and the mixture was reacted at 50 °C for 30 min. A 2.5 mL volume of 10% (*w*/*v*) trichloroacetic acid was added to the reaction mixture, followed by centrifugation at 3000 rpm for 10 min. A 1 mL volume of supernatant was added to a test tube and 1 mL of distilled water and 0.2 mL of 0.1% (*w*/*v*) FeCl_3_ solution were added to measure the reducing power using absorbance values at 700 nm.

### 3.11. Nitrite Scavenging Ability

Nitrite scavenging activity was measured by modifying the method of Kato et al. [43]. To 1 mL of 1 mM NaNO_2_ solution, 1 mL of the sample extract was added. The pH was adjusted to 1.2, 3.0, and 4.2 using 0.1 N HCl and 0.2 M citrate buffer solution, respectively, and the total volume was adjusted to 10 mL. These solutions were reacted at 37 °C for 1 h and then, 3 mL of 2% (*v*/*v*) acetic acid and 0.4 mL of Griess reagent (1% (*w*/*v*) sulfanilic acid: 1% (*w*/*v*) naphthylamine = 1:1) dissolved in 30% (*v*/*v*) acetic acid solution were added to 1 mL of each reaction mixture. After incubation at room temperature for 15 min, the absorbance was measured at 520 nm. The control group was measured by adding distilled water instead of Griess reagent, and the nitrite scavenging activity was calculated according to the following Equation (4):

(4) $$\mathrm{Nitrite}\;\mathrm{scavenging}\;\mathrm{activity}\;\left(\%\right)=\left(1-\left(\frac{Asample}{Acontrol}\right)\right)\times 100$$

where, A*sample*: absorbance of the sample added group; A*control*: absorbance of the no sample added group.

### 3.12. Organic Acid Analysis

Onion vinegar fermentation was evaluated by high performance liquid chromatography (HPLC) (Young Lin Instrument Co., Ltd., YL9100 HPLC system, Anyang, Korea) after filtration of the samples through a 0.45-μm membrane (Millipore). Lactic acid, acetic acid, succinic acid, malic acid, and citric acid dissolved in 0.1% (*w*/*v*) perchloric acid solution were used as reference materials. The retention time and peak area of these standards were used to calculate the organic acid content of the fermentation broth. HPLC conditions were modified from and supplemented by the method of Huang et al. [44] and a Shodex RSpak KC-811 (I.D. 8.0 × 3.00 mm) column (Showa Denko K.K., Tokyo, Japan) was used. The oven temperature, mobile phase, flow rate, injection volume, and detector conditions were 4 °C, 0.1% perchloric acid, 1.0 mL/min, 10 μL and UV 210 nm, respectively.

### 3.13. Sensory Evaluation

The sensory evaluation of the prepared vinegar was carried out by 15 panelists. The panelists were instructed on the purpose and evaluation method of this experiment, and then provided with 15 mL samples (four times diluted with drinking water). Scoring tests were used for the appearance, color, flavor, taste and overall acceptability of onion vinegar, with apple cider vinegar from O company (Seoul, Korea) (diluted seven times with drinking water) used as a control. The scores were rated as very good (5 points), good (4 points), average (3 points), poor (2 points), and very poor (1 point).

### 3.14. Statistical Analysis

Statistical analysis was performed with Statistical Analysis System (SAS) software (version 9.2) (SAS Institute Inc., Cary, NC, USA) [45]. After analysis of variance (ANOVA), Duncan’s multiple range test at *p* < 0.05 was used to determine the significance of the mean values among the experimental groups.

## 4. Conclusions

*Acetobacter orientalis* MAK88 isolated from Korean traditional rice wine harbored good properties for the fermentation of vinegar using onion, such as high acid production, good growth rate in onion juice, and high acid- and alcohol-tolerance. Semi-continuous fermentation of onion juice with three cycles of charge-discharge method significantly reduced the fermentation time and simplified the vinegar production process. Onion vinegar fermented in this study showed higher functional properties, including antioxidant activities and higher organoleptic acceptability than commercial onion vinegar. In an aspect of agricultural economics, this study presented a way to utilize surplus onion which otherwise would be disposed of as agricultural waste.

## Figures and Tables

**Table 1:** Parameter estimates after two-stage onion vinegar fermentation and semi-continuous onion vinegar fermentation.

Parameter	Oxygen Supply Rate (vvm) for Two-Stage Fermentation			Cycle of Semi-Continuous Fermentation		
	0.2	0.5	1	1	2	3
Y_A/E_ (%) *	65.47	60.33	61.09	66.97	67.81	76.71
P (g/(L·d)) **	10.75	9.30	9.45	13.41	12.53	17.73
Π (g/(g·h)) ***	18.78	16.32	10.36	18.57	18.80	20.58

*: $Fermentatoin\;efficiency=\frac{final\;acidity\;\left(\%\right)\;-\;initial\;acidity\;\left(\%\right)}{\left(initial\;ethanol\;concentration\;\left(\%\right)\;-\;final\;ethanol\;concentration\;\left(\%\right)\right)\;\times \;1.304}\times 100$; **: $Acetic\;acid\;productivity=\frac{final\;acidity\;\left(g/L\right)\;-\;initial\;acidity\;\left(g/L\right)}{fermentation\;time\;\left(day\right)}$; ***: $Specific\;production\;rate=\frac{final\;acidity\;\left(\%\right)\;-\;initial\;acidity\;\left(\%\right)}{\left(final\;dry\;cell\;weight\;-\;initial\;dry\;cell\;weight\right)}$.

**Table 2:** Nitrite-scavenging activity of onion vinegar and various commercial vinegars.

pH	Sample	Scavenging Activity (%)
1.2	Onion vinegar (in this study)	95.38 ± 3.63 ^b,^*
	Commercial onion vinegar	61.99 ± 2.20 ^d^
	Commercial apple vinegar	14.62 ± 0.49 ^e^
	Commercial pomegranate vinegar	89.68 ± 1.96 ^c^
	10 mM ascorbic acid	99.06 ± 0.78 ^a^
3	Onion vinegar (in this study)	49.37 ± 4.29 ^a^
	Commercial onion vinegar	21.43 ± 0.84 ^c^
	Commercial apple vinegar	11.43 ± 0.84 ^d^
	Commercial pomegranate vinegar	48.78 ± 4.71 ^a^
	10 mM ascorbic acid	38.44 ± 2.57 ^b^
4.2	Onion vinegar (in this study)	29.32 ± 6.63 ^a^
	Commercial onion vinegar	8.54 ± 3.93 ^b^
	Commercial apple vinegar	0.62 ± 0.16 ^c^
	Commercial pomegranate vinegar	8.70 ± 3.45 ^b^
	10 mM ascorbic acid	23.20 ± 1.96 ^a^

* Different *superscripts* are indicative of a statistically significant difference (*p* < 0.05).

**Table 3:** Organic acid content in onion vinegar.

Organic Acid	Concentration (g/L)	Percentage (%)
Acetic acid	36.85 ± 0.15	87.41
Citric acid	4.94 ± 0.03	11.71
Malic acid	0.37 ± 0.00	0.88
Lactic acid	ND *	ND
Succinic acid	ND	ND

* ND: Not detected.
